# Taxonomy, Phylogenetic and Ancestral Area Reconstruction in *Phyllachora*, with Four Novel Species from Northwestern China

**DOI:** 10.3390/jof8050520

**Published:** 2022-05-18

**Authors:** Jin-Chen Li, Hai-Xia Wu, Yuying Li, Xin-Hao Li, Jia-Yu Song, Nakarin Suwannarach, Nalin N. Wijayawardene

**Affiliations:** 1 International Fungal Research and Development Centre, Institute of Highland Forest Science, Chinese Academy of Forestry, Kunming 650224, China; jinchenlii@163.com (J.-C.L.); lixinhao91537@gmail.com (X.-H.L.); ldsat-f@outlook.com (J.-Y.S.); 2 Key Laboratory of Breeding and Utilization of Resource Insects, National Forestry and Grassl and Administration, Kunming 650224, China; 3 College of Grassland Science, Shanxi Agricultural University, Mingxian South Road, Taigu District, Jinzhong 030801, China; liyuy_ing@sxau.edu.cn; 4 Research Center of Microbial Diversity and Sustainable Utilization, Chiang Mai University, Chiang Mai 50200, Thailand; suwan.462@gmail.com; 5 Centre for Yunnan Plateau Biological Resources Protection and Utilization, College of Biological Resource and Food Engineering, Qujing Normal University, Qujing 655011, China; nalinwijayawardene@yahoo.com; 6 Section of Genetics, Institute for Research and Development in Health and Social Care, No. 393/3, Lily Avenue, Off Robert Gunawardane Mawatha, Battaramulla 10120, Sri Lanka

**Keywords:** Ancestral reconstruction, Multigene phylogeny, Morphology, four new taxa, *Phyllachora*

## Abstract

The members of *Phyllachora* are biotrophic, obligate plant parasitic fungi featuring a high degree of host specificity. This genus also features a high degree of species richness and worldwide distribution. In this study, four species occurring on leaf and stem of two different species of grass were collected from Shanxi and Shaanxi Provinces, China. Based on morphological analysis, multigene (combined data set of LSU, SSU, and ITS) phylogenetic analyses (maximum likelihood and Bayesian analysis), and host relationship, we introduce herein four new taxa of *Phyllachora*. Ancestral area reconstruction analysis showed that the ancestral area of *Phyllachora* occurred in Latin America about 194 Mya. Novel taxa are compared with the related *Phyllachora* species. Detailed descriptions, illustrations, and notes are provided for each species.

## 1. Introduction

Phyllachorales is an ascomycetous order (in Sordariomycetes) introduced by Barr [1]. This order comprises biotrophic, obligate plant parasitic fungi that infect mostly plant leaves and stems [2,3,4]. Species of Phyllachorales are mainly distributed in tropical and subtropical regions [5,6,7,8]. Members of Phyllachorales present with shiny black stromata, leading to the common name ‘tropical tar spot fungi’. Most of the members of Phyllachorales are parasitic on angiosperms with a few notable exceptions including the lichenicolous *Lichenochora* species, the marine algicolous genus *Phycomelaina*, as well as ferns and gymnosperms [8,9,10,11].

Currently, Phyllachorales has four families, including Phaeochoraceae, Phaeochorellaceae, Phyllachoraceae, and Telimenaceae [12,13]. They are morphologically characterized by black stromata of various shapes in the host plant; having paraphyses; unitunicate asci cylindrical to clavate in shape, with an inconspicuous apical ring, usually 8-spored; and aseptate ascospores, which in most species are hyaline and 1-celled, appearing as brown in a few species (e.g., *Phyllachora stenostoma*) [1,4,6,8,14]. The asexual morph of Phyllachorales has been reported as a coelomycetous morph [15]. Large-scale phylogenetic studies comprising many representative species have confirmed the position of Phyllachorales in the subclass Sordariomycetidae with high support (100% MLBP) as well as the monophyly of the order [4,8,16]. Mardones et al. [8] used three morphological characteristics and one ecological characteristic to reconstruct the ancestral state of genera in Phyllachorales based on the Likelihood Ancestral States method, reasoning that these characteristics had evolved independently numerous times. The ancestral state of members of Phyllachorales were monocotyledonous host plants with immersed perithecia, which was lost in the family Phaeochoraceae and evolved into erumpent or superficial perithecia in some species of Phyllachoraceae. The presence of clypeus as a morphological characteristic was lost only once in Phaeochoraceae. Therefore, it is thought that the presence of clypeus in these fungi is an evolutionarily stable characteristic. The ancestor of the Phyllachorales species had a black stroma, and the presence of bright black stromata may have evolved at least twice.

The family Phyllachoraceae was introduced by Theissen and Sydow [17] with *Phyllachora* as the type genus [3,18]. It is the largest family in Phyllachorales and currently comprises 54 genera [13]. Members of the family are characterized by forming leaf spots on the host that are abundant but scattered, raised, mostly rounded to oblong or elongated, sometimes parallel with leaf venation, surrounded by a light-brown necrotic region; lacking periphyses; having numerous paraphyses, branched or unbranched; 8-spored asci, persistent, cylindrical to fusiform, often present with an apical ring; ascospores fusiform to narrowly oval, hyaline, often with a mucilaginous sheath [4]. The type genus, *Phyllachora*, was introduced based on *P. agrostis*, which is a single species on the herbarium label in Fuckels exsiccate series ‘Fungi Rhenani’ [5]. Phyllachoraceae is similar to Phaeochoraceae, but Phaeochoraceae species are characterized by 6-8-spored asci, usually without apical structure, yellow to olivaceous ascospores or in various shades of brown, thick-walled; conversely, Phyllachoraceae species are characterized by 8-spored asci, an often-present an apical ring, usually hyaline ascospores, rarely pale brown, thin and smooth-walled [4,8]. These morphological characteristics can be used to distinguish the two families, and they form two independent branches in the phylogenetic tree [8].

*Phyllachora* is the type genus of Phyllachoraceae. Clements [19] designated the lectotype as *Phyllachora graminis*. Currently, *Phyllachora* is the largest genus within Phyllachoraceae, and about 1513 epithets are listed in the Index Fungorum (Index Fungorum 2022; accession date: 28.03.2022). Nevertheless, only 1382 species are accepted in the Species Fungorum (accession date: 28.03.2022). Species of the genus are morphologically characterized by clypeate pseudostroma in leaf tissues; generalized infection of the entire section of the mesophyll forming leaf spots on the host, mostly rounded to oblong or elongated, surrounded by a light-brown necrotic region; perithecium globose; numerous paraphyses, branched, slightly longer than asci; asci 8-spored, persistent, cylindrical to fusiform, short pedicellate, an apical ring often present; and ascospores 1–3 seriate, fusiform to narrowly oval, hyaline, sometimes with a gelatinous sheath [4,18,20]. Some members of the genus can inflict crop diseases, leading to yield loss. *Phyllachora maydis* is an example occurring in the United States, which can seriously impact quality and corn yield [21,22,23,24]. Owing to its biotrophic habit and high degree of host specificity, most *Phyllachora* species are given names based on host association and coevolution with the host [5,8,20,25]. *Phyllachora* species cannot grow on agar media since they are biotrophic [8]. *Phyllachora* species have been reported as pathogenic species on more than 1000 plant species (belonging to 121 families, including Cyperaceae, Fabaceae, Lauraceae, Moraceae, Myrtaceae, Poaceae, Proteaceae, and Rosaceae), and they are commonly found with Poaceae [20,26,27].

In this study, several specimens with tar spot diseases were collected. Based on polyphasic approaches (e.g., morphological analyses, information of host plant, and phylogenetic analyses), four novel species of *Phyllachora* are introduced herein. Based on paleontological evidence and paleoclimate records, we also reconstructed the ancestral area of *Phyllachora*. The analysis was restricted to members of *Phyllachora*, considering the history of their biogeographic diversity and dispersal route as well as estimating the divergence time and ancestral location of this genus.

## 2. Materials and Methods

### 2.1. Collecting, Morphological Study, and Depositing Specimens

Phyllachora-like fungi were collected from living leaves of *Cenchrus flaccidus* (Poaceae) and *Chloris virgata* (Poaceae) during field surveys in 2019 in Shanxi and Shaanxi Provinces, China. Specimens were taken to the laboratory in paper envelopes. Specimens were processed and examined with microscopes, and photos of ascomata and host were taken using a compound stereomicroscope (KEYENCE CORPORATION V.1.10 with camera VH-Z20R) following Wu et al. [28]. Hand sections were made under a stereomicroscope (OLYMPUS SZ61) and mounted in water and blue cotton, and photomicrographs of fungal structures were taken with a compound microscope (Nikon ECLIPSE 80i). 

Images used for figures were processed with Adobe Photoshop CC v. 2015.5.0 software (Adobe Systems, San Jose, CA, USA).

Holotype collections were deposited at the herbarium of IFRD (International Fungal Research & Development Centre; Institute of Highland Forest Science, Chinese Academy of Forestry, Kunming, China). Registration numbers for new species were obtained in MycoBank Database (https://www.mycobank.org/, accession date: 10 April 2022).

For our specimens, no culture was obtained by multiple single-spore isolation or tissue isolation.

### 2.2. DNA Isolation, Amplification, and Sequencing

In accordance with the manufacturer’s instructions, genomic DNA was extracted from ascomata at room temperature using the Forensic DNA Kit (OMEGA, New York, NY, USA). The primers LR0R and LR5 were used to amplify the large subunit (LSU) rDNA [29]. The internal transcribed spacer (ITS) rDNA was amplified and sequenced with the primers ITS5 and ITS4 [30]. The partial small subunit (SSU) rDNA was amplified using primers NS1 and NS4 [30]. PCR reactions were in accordance with instructions from Golden Mix, Beijing TsingKe Biotech Co. Ltd, Beijing, China: initial denaturation at 98 °C for 2 min, followed by 30 cycles of 98 °C denaturation for 10 s, 56 °C annealing for 10 s and 72 °C extensions for 10 s (ITS and SSU) or 20 s (LSU), and a final extension at 72 °C for 1 min. All PCR products were sequenced by Biomed (Beijing Biomed Gen Technology Co., Ltd., Beijing, China). PCR products were sequenced by Biomed using the same primers as before.

### 2.3. Sequence Alignment and Phylogenetic Analyses

BioEdit version 7.0.5.3 [31] was used to re-assemble the sequences generated from forward and reverse primers for obtaining integrated sequences. Sequences of Phyllachoraceae species were downloaded from GenBank (Table 1) following the relevant publications [8,20,32,33]. All sequences were adjusted manually with Bioedit 7.0.5.3 [31] and aligned using the default setting of MAFFT version 7 online [34] (https://mafft.cbrc.jp/alignment/server/, accession date: 10 April 2022). 

Maximum Likelihood (ML) analysis using the aligned sequences as input was conducted with the help of RAxNLGUI v. 2.0 [35]. *Telimena bicincta* (MM-108) and *T. bicincta* (MM-133) were selected as an outgroup. One thousand nonparametric bootstrap iterations were employed with the “ML + rapid bootstrap” tools and “GTRGAMMA” arithmetic.

For Bayesian analysis, MrModeltest 2.3 [36] was used to estimate the best-fitting model for the combined LSU, SSU, and ITS loci, and model GTR+G was the best fit. In MrBayes v.3.2 [37], six simultaneous Markov chains were run for 2,000,000 generations; trees were sampled and printed every 100 generations. The first 5000 trees were submitted to the burn-in phase and discarded, while the remaining trees were used for calculating posterior probabilities in the majority rule consensus tree [38,39,40,41].

### 2.4. Reconstruction of Ancestral State

Members of *Phyllachora* were coded based on their collection locality according to field notes and references. Six areas were delimited based on the distribution data of *Phyllachora*: A = East Asia, B = Southeast Asia, C = North America, D = South America, E = Latin America, F = Central Europe, G = Unknown, using species from Asia, Europe, North America, South America, Latin America, and Central Europe. In MrBayes v.3.2, chains were run for 1 00000 generations; trees were sampled and printed every 100 generations. RASP 4.2 (Reconstruct Ancestral State in Phylogenies, http://mnh.scu.edu.cn/soft/blog/RASP, accession date: 10 April 2022) was used to reconstruct the ancestral state, and the most-optimal model was DEC [42].

### 2.5. Calibration Procedure

The second calibration time referenced the results of Dayarathne et al. [33] and Hongsanan et al. [43]. We followed the conclusion that the family Phyllachoraceae divergence time was about 217 Mya as a calibration point (root) for ancestral distribution reconstruction.

## 3. Results

### 3.1. Molecular Phylogenetic Results

**Figure 1:**
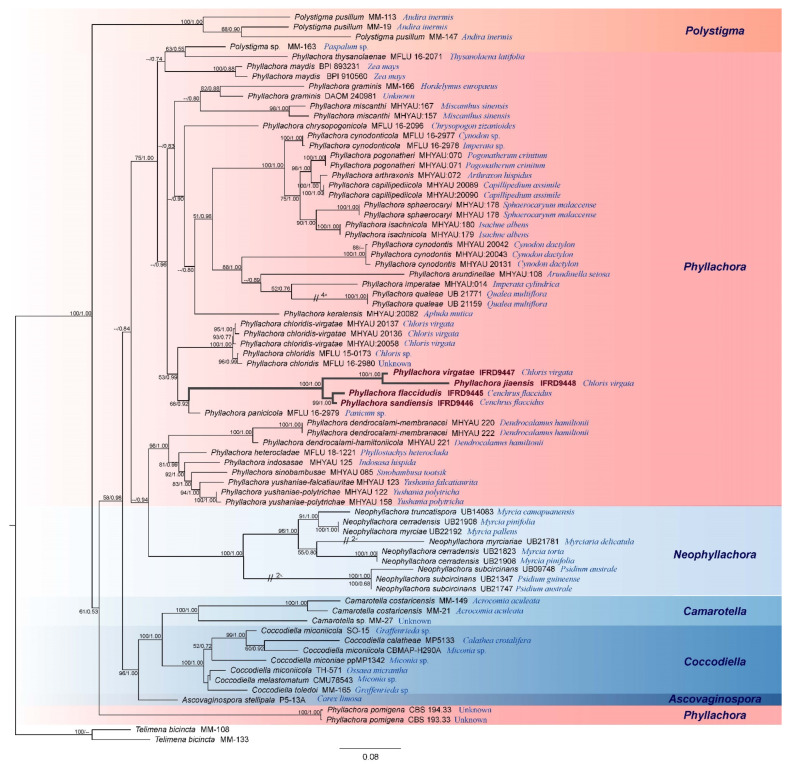
Phylogenetic tree of maximum likelihood showing the relationships of Phyllachoraceae based on combined LSU, SSU, and ITS data set analysis. Bootstrap values of maximum likelihood higher than 50% are shown on the left, while values of Bayesian posterior probabilities above 0.5 are shown on the right. New species are given in bold, followed by the host of the species behind its strain number.

We analyzed a three-loci (LSU, SSU, ITS) data set of *Phyllachora*. Based on the combined data of LSU, SSU, and ITS sequences. It was found that the two topological trees obtained by maximum likelihood (ML) and Bayesian were similar, and the best scoring RAxML tree was used as the representative tree (Figure 1). We generated a total of 161 sequences from 74 taxa of Phyllachorales, 57 sequences of LSU, 34 sequences of SSU, 70 sequences of ITS, and concatenated sequences of three genes, with 3341 characters including gaps. Bootstrap values of ML higher than 50% are shown on the phylogenetic tree, while values of Bayesian posterior probabilities above 0.5 are shown on the tree (Figure 1). Phylogenetic analysis showed that all four new taxa belonging to *Phyllachora* cluster together with *Phyllachora panicicola* with bootstrap values of 68% (in ML analysis) and Bayesian posterior probability of 0.92. *Phyllachora panicicola* and four new taxa form two clades independent from each other with bootstrap values of 100% and Bayesian posterior probabilities of 1.00.

### 3.2. Ancestral Area Reconstruction Analysis for Phyllachora

**Figure 2:**
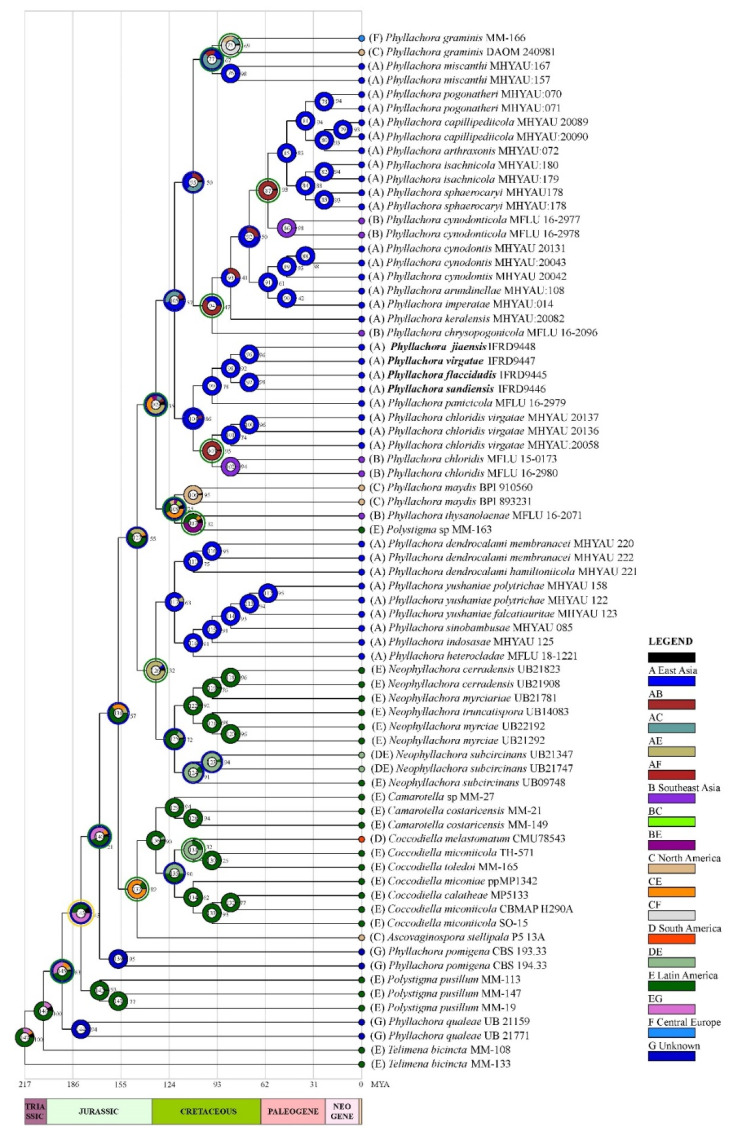
Ancestral character state reconstruction based on the Bayesian tree. Each event is represented with a number at the nodes. Bayesian posterior probabilities are presented (≥0.5). The colored circle near the number at the nodes indicate that blue represents Dispersal, green represents Vicariance, orange represents Extinction. New species are given in bold.

Ancestral area reconstruction analysis revealed that *Phyllachora* species originated from Latin America about 194 Mya (Figure 2, node 145). Dispersal, vicariance, extinction, and other historical events affected the biogeographical distribution of the species. The evolutionary history of ancestors from the genus *Phyllachora* reveals that the species of this genus underwent 20 dispersals, 13 vicariances, and 1 extinction (Figure 2, blue coils represent dispersal, green coils represent vicariance, and orange coils represent extinction). Species of *Phyllachora* migrated from Latin America to Southeast Asia during the Jurassic period, with two dispersal events noted (Figure 2, node 127). In approximately 60–155 Mya, there were frequent dispersal and vicariance events, and moreover, vicariances were always accompanied by dispersal events. There is only low support suggesting that species belonging to *Phyllachora* may have migrated from Latin America to Southeast Asia 119 Mya (Figure 2, node 108). About 100 Mya, species migrated from East Asia or North America to central Europe, with one dispersal and one vicariance (Figure 2, node 77).

### 3.3. Taxonomy of Fungi

**Figure 3:**
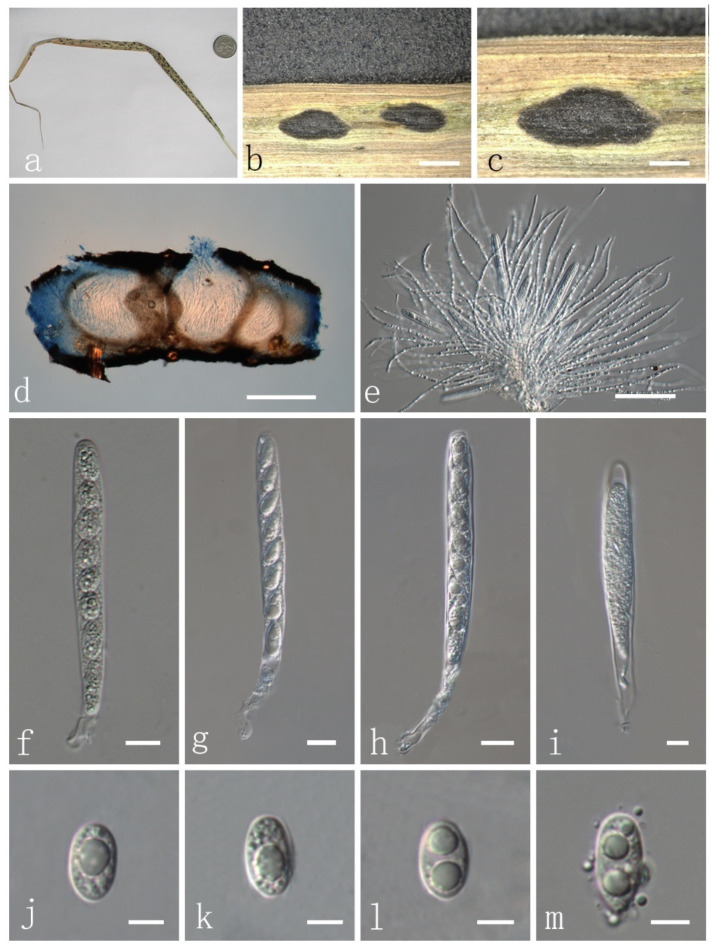
*Phyllachora flaccidudis* (IFRD9445, holotype). (**a**) Black spots on *Cenchrus flaccidus* (Poaceae); (**b,c**) Stromata; (**d**) Vertical section of ascomata in cotton blue; (**e**) Paraphyses; (**f**–**i**) Asci; (**j**–**m**) Ascospores. Scale bars, (**b**) 1 mm; (**c**)0.5 mm; (**d**) 200 μm; (**e**) 50 μm; (**f**–**i**) 10 μm; (**j**–**m**) 5 μm. Microscopic techniques: DIC.

**Figure 4:**
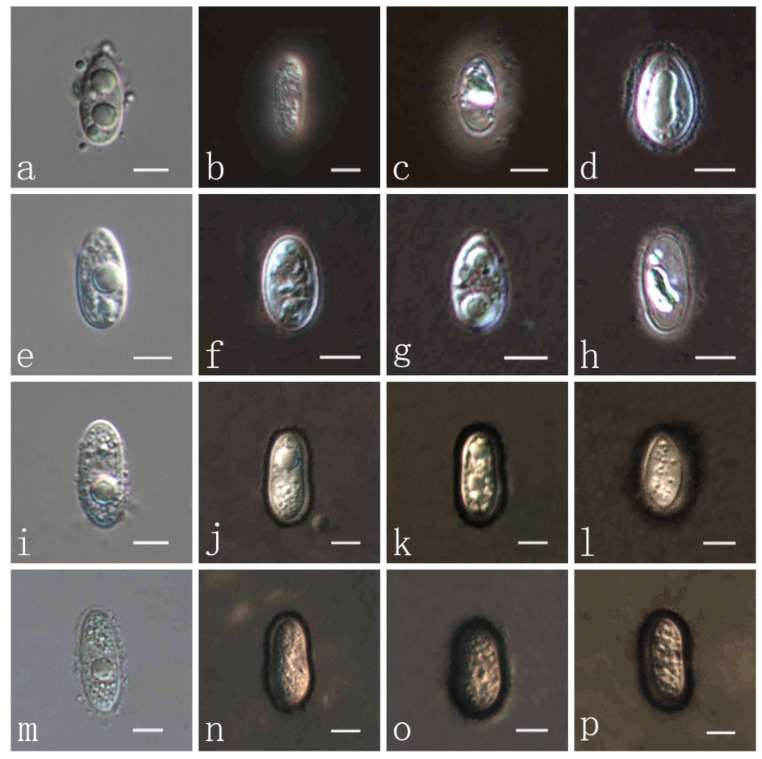
(**a**) *P. flaccidudis* ascospore in DIW (Deionized Water); (**b**,**c**) *P. flaccidudis* ascospores with gelatinous sheath in ink; (**e**) *P. sandiensis* ascospore in DIW; (**f**–**h**) *P. sandiensis* ascospores with gelatinous sheath in ink; (**i**) *P. virgatae* ascospore in DIW; (**j**,**k**) *P. virgatae* ascospores with gelatinous sheath in ink; (**m**) *P. jiaensis* ascospore in DIW; (**n**–**p**) *P. jiaensis* with gelatinous sheath in ink. Scale bars, (**a**–**p**) 5 μm. Microscopic techniques: DIC.

***Phyllachora flaccidudis*** H. X. Wu & J. C. Li. sp. nov. (Figure 3 and Figure 4).

MycoBank: MB843395.

*Etymology:* Epithet derived from host species *Cenchrus flaccidus*.

Holotype: IFRD9445.

Parasitic on leaves and stems of *Cenchrus flaccidus* (Poaceae). Sexual morph: Stroma 1618–1900 × 641–764 μm ($\bar{x}$ = 1769 × 716 μm, *n* = 10) (Figure 3a–c), fusiform or cymbaeform, domed above the leaf surface, amphigenous, scattered, sometimes gregarious, like black nevus, no edge, black, carbonaceous. Section of stroma 307–379 μm high, multilocular, peridium 35–60 μm wide, composed of brown to dark brown cells of textura angularis (Figure 3d). Paraphyses 2–3 μm wide ($\bar{x}$ = 2.9 μm, *n* = 20), numerous, persistent, filiform, unbranched, aseptate, many guttules, slightly longer than asci (Figure 3e). Asci 82–110 × 7–10 μm ($\bar{x}$ = 91.6 × 9.0 μm, *n* = 20), thin-walled, 8-spored, persistent, cylindrical to clavate, apex obtuse, with pedicel (Figure 3f–i). Ascospores 11–13 × 4–7 μm ($\bar{x}$ = 11.4 × 5.9 μm, *n* = 20), 1-seriate, fusiform to oval, both ends obtuse, hyaline, aseptate, verrucose, with many guttules (Figure 3j–m) and a mucilaginous sheath (Figure 4a–d). Asexual morph: Not observed.

Material examined: China, Shanxi Province, Xinzhou, Wutai County, 38.71917699 N, 113.25921 E, on stems and leaves of *Cenchrus flaccidus* (Poaceae), 4 October 2019, Yuying Li IFRD9445, holotype. GenBank accession numbers: ITS: ON075524, LSU: ON072101, SSU: ON072097.

Notes: *Phyllachora flaccidudis* was collected from *Cenchrus flaccidus* (Poaceae) in Shanxi Province of China. According to the phylogenetic analysis, *P. flaccidudis* and *P. sandiensis* are closely related to *P. panicicola* (Figure 1). However, *P. panicola* was reported from *Panicum* sp., and its asci were significantly longer and wider than in *P. flaccidudis* and *P. sandiensis* (110–130 × 10–14 μm vs. 82–110 × 7–10 μm, 92–126 × 7–10 μm, respectively). Furthermore, the ascospores of *P. panicola* are significantly larger than in *P. flaccidudis* and *P. sandiensis* (14–16 × 6–8 μm vs. 11–13 × 4–7 μm, 10–14 × 6–7 μm, respectively). The ascospores of *P. panicicola* are rounded at both ends, with a central concave depression and lacking guttules different from *P. flaccidudis* and *P. sandiensis*. Differences in morphological characteristics are also supported by the phylogenetic tree, as the two species cluster independently (100% bootstrap support) in a subclade of *Phyllachora* (Figure 1).

The hosts of *Phyllachora sphaerosperma* (= *Phyllachora cenchricola*), *P. flaccidudis* and *P. sandiensis* belong to species of *Cenchrus*. However, the host of *P. cenchricola* is *Cenchrus echinatus*, which has been found in Brazil, the southern United States, South America, and the West Indies. The ascospores are nearly spherical, wider than both *P. flaccidudis* and *P. sandiensis* (Table 2).

*Phyllachora flaccidudis* and *P. chloridis* [33] have similar morphological characteristics, but their host plants are different. The host of *P. flaccidudis* and *P. sandiensis* was reported from *Cenchrus flaccidus* (Poaceae), while the host of *P. chloridis* is *Chloris* sp. Morphologically, the asci of *P. flaccidudis* and *P. sandiensis* are significantly longer than those of *P. chloridis* (Table 2) and feature pedicels, but they are absent in *P. chloridis*. Ascospores of *P. flaccidudis* and *P. sandiensis* have 1–2 or more guttules, while *P. chloridis* has only one central guttule, which can serve as an important characteristic for species delimitation.

**Figure 5:**
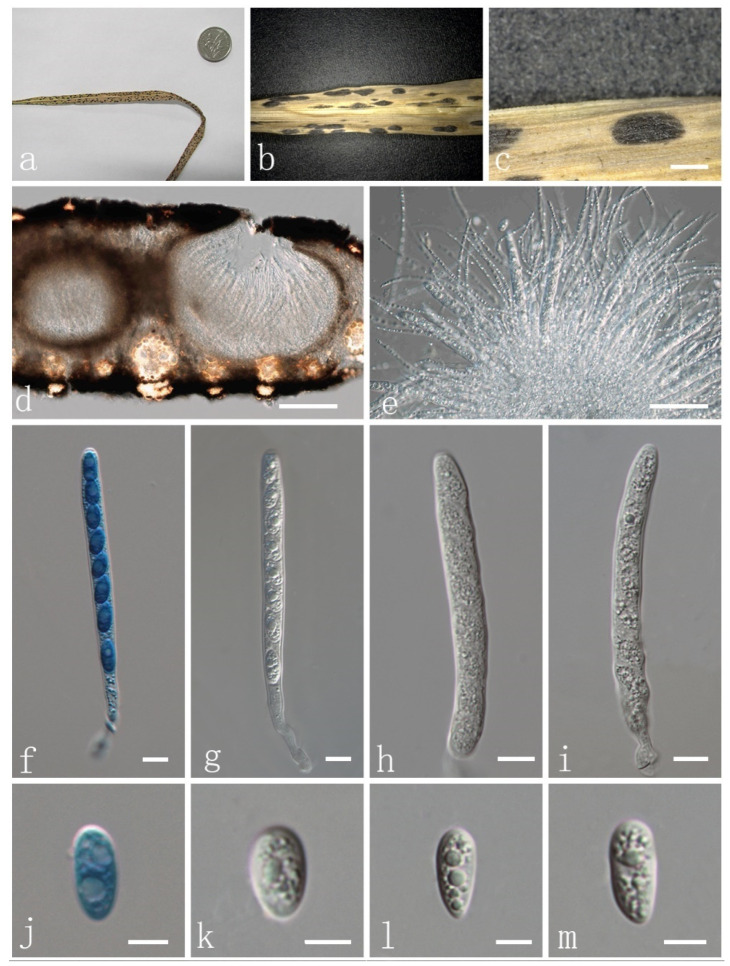
*Phyllachora sandiensis* (IFRD9446, holotype). (**a**) Black spots on *Cenchrus flaccidus* (Poaceae); (**b**,**c**) Stromata; (**d**) Vertical section of ascomata; (**e**) Paraphyses; (**f**) Ascus in cotton blue; (**g**–**i**) Asci; (**j**) Ascospore in cotton blue; (**k**–**m**) Ascospores. Scale bars, (**c**) 0.5 mm; (**d**) 100 μm; (**e**) 50 μm; (**f**–**i**) 10 μm; (**j**–**m**) 5 μm. Microscopic techniques: DIC.

***Phyllachora sandiensis*** H. X. Wu & J. C. Li. sp. nov. (Figure 4 and Figure 5).

MycoBank: MB 843396.

*Etymology:* Epithet derived from the type locality, a sandy forest park roadside (Shaanxi Province, Yulin City, Yuyang District) in China.

Holotype: IFRD9446.

Parasitic on leaves and stems of *Cenchrus flaccidus* (Poaceae). Sexual morph: Stroma 993–2742 × 371–438 μm diam. ($\bar{x}$ = 1378 × 415 μm, *n* = 10) (Figure 5a–c), domed above the leaf surface, amphigenous, fusiform, cymbaeform or irregular shape, like black nevus, scattered, sometimes gregarious, no edge, black, carbonaceous. Section of stroma 344–421 μm high, oval, multilocular, peridium 36–58 μm wide, composed of brown to dark-brown cells of textura angularis (Figure 5d). Paraphyses 2–4 μm wide ($\bar{x}$ = 3 μm, *n* = 20), numerous, persistent, filiform, unbranched, aseptate, many guttules, slightly longer than asci (Figure 5e). Asci 92–126 × 7–10 μm ($\bar{x}$ =106.6 × 9.7 μm, *n* = 20), thin–walled, 8-spored, persistent, cylindrical to clavate, apex obtuse, with pedicel (Figure 5f–i). Ascospores 10–14 × 6–7 μm ($\bar{x}$ =12.3 × 6.3 μm, *n* = 20), 1-seriate, droplet or oval to ellipse, acute at the end, narrowly rounded at another end, hyaline, aseptate, verrucose, with 1–3 guttules (Figure 5j–m) and a thin mucilaginous sheath (Figure 4e–h). Asexual morph: Not observed.

Material examined: China, Shaanxi Province, Yulin City, Yuyang District, sandy forest park roadside, 38.29470799 N, 109.75387 E, on stems and leaves of *Cenchrus flaccidus* (Poaceae), 7 October 2019, Yuying Li, IFRD9446, holotype. GenBank accession numbers: LSU: ON075528, SSU: ON072098, ITS: ON075525.

Notes: *Phyllachora sandiensis* was collected from *Cenchrus flaccidus* in Shaanxi Province of China. According to the phylogenetic analysis, *P. sandiensis* is closely related to *P. flaccidudis*, but the samples were collected from different locales. Morphologically, the size of asci and stromata of *P. flaccidudis* is significantly longer than in the case of *P. sandiensis* (Table 2). In addition, the ascospores of *P. sandiensis* are longer than those of *P. flaccidudis* (average 106.6 × 9.7 μm vs. 91.6 × 9.0 μm). According to sequence alignment results, LSU, SSU, and ITS sequences differed by 9 bases, 5 bases, and 13 bases between both taxa, respectively. Therefore, *P. sandiensis* is considered to be a new species of *Phyllachora*.

**Figure 6:**
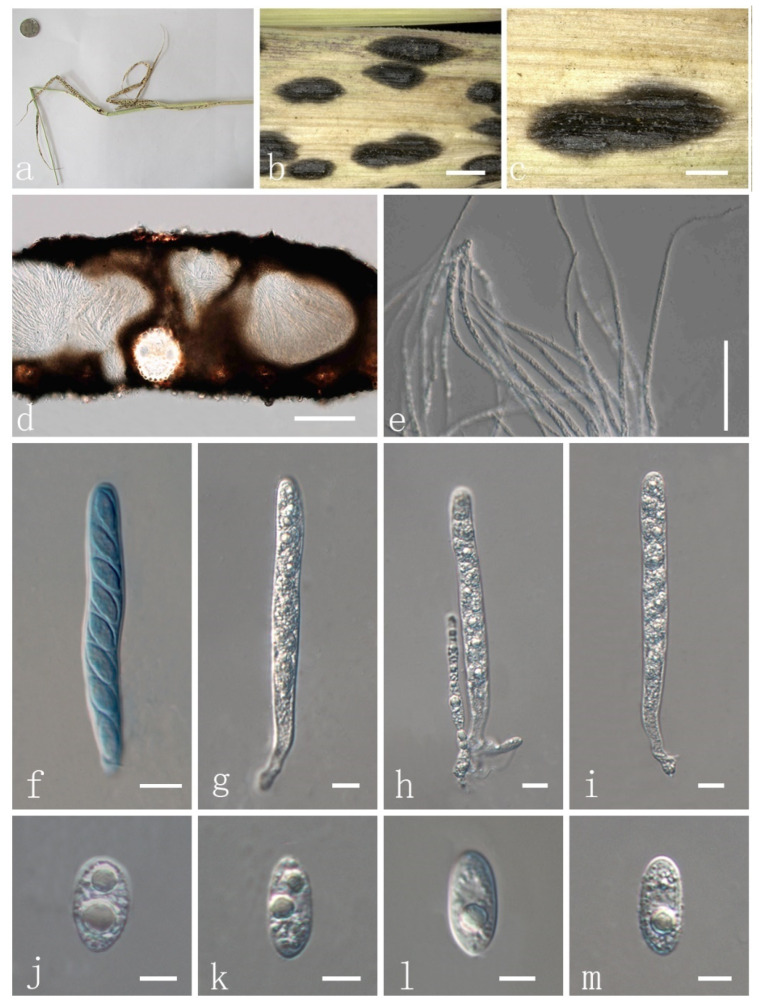
*Phyllachora virgatae* (IFRD9447, holotype). (**a**) Black spots on *Chloris virgata* (Poaceae); (**b**,**c**) Stromata; (**d**) Vertical section of ascomata; (**e**) Paraphyses; (**f**) Ascus in cotton blue; (**g**–**i**) Asci; (**j**–**m**) Ascospores. Scale bars, (**b**) 1 mm, (**c**) 0.5 mm; (**d**) 100 μm; (**e**) 20 μm; (**f**–**i**) 10 μm; (**j**–**m**) 5 μm. Microscopic techniques: DIC.

***Phyllachora virgatae*** H. X. Wu & J. C. Li. sp. nov. (Figure 4 and Figure 6).

MycoBank: MB843397.

*Etymology:* Epithet derived from host species *Chloris virgata*.

Holotype: IFRD9447.

Parasitic on leaves and stems of *Chloris virgata* (Poaceae). Sexual morph: Stroma 738–2678 × 490–701 μm ($\bar{x}$ = 1762 × 642 μm, *n* = 10) (Figure 6a–c), domed above the leaf surface, amphigenous, fusiform, cymbaeform or irregular shape, black spots, scattered, sometimes gregarious, no edge, shiny black, carbonaceous. Section of stroma 215–232 μm high, oval, multilocular, peridium 25–32 μm wide, composed of brown to dark-brown cells of textura angularis (Figure 6d). Paraphyses 2–3.5 μm wide ($\bar{x}$ = 3.3 μm, *n* = 20), numerous, persistent, filiform, unbranched, aseptate, slightly longer than asci (Figure 6e). Asci 84–120 × 7–11 μm ($\bar{x}$ = 102.6 × 9.1 μm, *n* = 20), thin-walled 8-spored, persistent, clavate, apex obtuse, with pedicel (Figure 6f–i). Ascospores 10–15 × 6–9 μm ($\bar{x}$ = 12.9 × 6.9 μm, *n* = 20), 1-seriate, ovoid to oblong, acute at both ends, hyaline, aseptate, verrucose, with 1–2 or more guttules (Figure 6j–m) and a glutinous mucilaginous sheath (Figure 4i–l). Asexual morph: Not observed.

Material examined: China, Shanxi Province, Xinzhou, Dingxiang County, 38.49269099 N, 112.94377 E, on stems and leaves of *Chloris virgata* (Poaceae), 4 October 2019, Yuying Li, IFRD9447, holotype. GenBank accession numbers: LSU: ON075439, SSU: ON072099, ITS: ON075526.

Notes: In the course of investigating the grass resources of northern China, two fungal species were collected from the *Chloris virgata* in Shanxi and Shaanxi Provinces. According to phylogenetic analysis, these two species are related to *P. panicicola*. They can be easily distinguished from *P. panicicola* based on a different host plant, larger asci (110–130 × 10–14 μm vs. 84–120 × 7–11 μm, 77–114 × 8–12 μm), and ascospore size (14–16 × 6–8 μm vs. 10–15 × 6–9 μm). Furthermore, the ascospores of *P. panicicola* are lacking guttules and have one central concave depression, differing from *P. virgatae* and *P. jiaensis*. 

*Phyllachora cynodontis* and *P. koondrookensis* have been reported from the same host (i.e., *Chloris*) [14,46]. However, *P. virgatae* and *P. jiaensis* are clearly distinguishable from the two species (Table 2). *Phyllachora chloridis-virgatae* (MHYAU 20136), *P. chloridis-virgatae* (MHYAU 20137), and *P. chloridis-virgatae* (MHYAU 20058) all have *Chloris virgata* as a host species, and there are no references about their morphological characteristics. However, they did not cluster with *P. virgatae* and *P. jiaensis* in the phylogenetic analysis.

We also searched for *Phyllachora* species reported from the same host genus in Farr et al. [49]. The results showed that *Phyllachora africana* (= *P. oblongospora*), *P. graminis*, and *P. minutissima* (=*P. bonariensis*) can also be parasitic on *Chloris* species. *Phyllachora chloridis*, *P. graminis*, *P. africana*, *P. minutissima*, *P. virgatae*, and *P. jiaensis* have been reported from the same host genus. However, morphologically they are easily distinguishable: the asci and ascospores of *P. virgatae* and *P. jiaensis* were significantly longer than in *P. chloridis*, *P. graminis*, and *P. minutissima* (Table 2). However, the asci of *P. chloridis* lack pedicels, and the asci of *P. graminis* have an ascus crown at the apex. *Phyllachora minutissima* has no paraphyses, while *P. virgatae* and *P. jiaensis* have pedicels and paraphyses and lack an ascus crown at the apex. The ascospores of *P. virgatae* and *P. jiaensis* have 1–2 or more guttules, but *P. chloridis* has only one central guttule. The asci of *P. africana* Parbery and *P. minutissima* were significantly longer than in *P. virgatae* and *P. jiaensis*. Hence, *P. virgatae* is distinguishable by its different morphological characteristics, which qualify it as a new species of *Phyllachora*.

**Figure 7:**
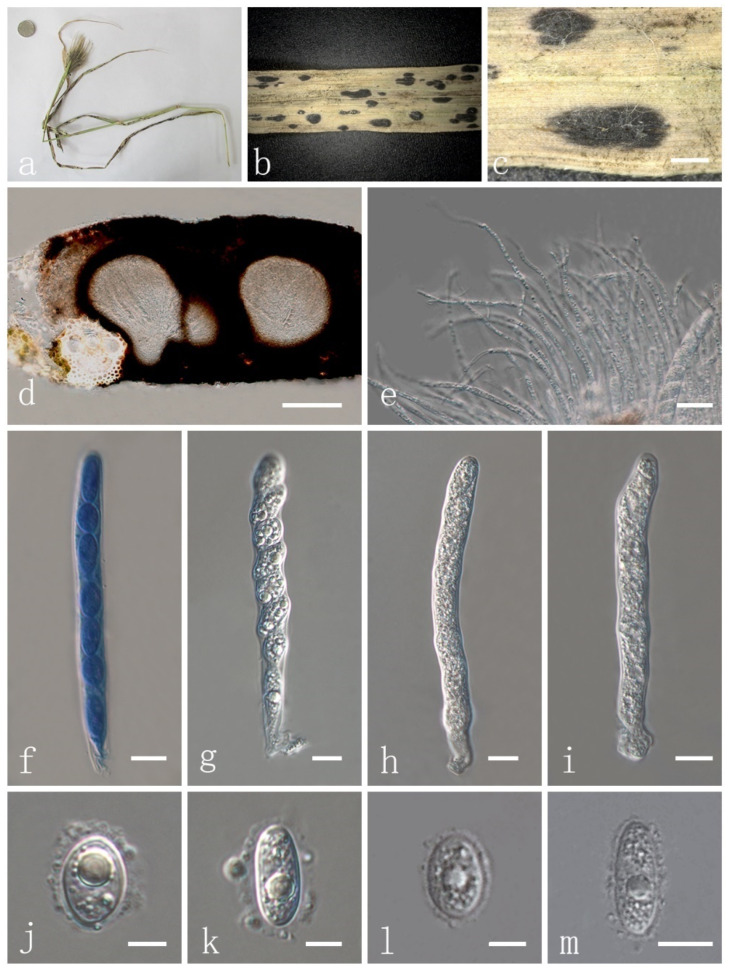
*Phyllachora jiaensis* (IFRD9448, holotype). (**a**) Black spots on *Chloris virgata* (Poaceae); (**b**,**c**) Stromata; (**d**) Vertical section of ascomata; (**e**) Paraphyses; (**f**) Ascus in cotton blue; (**g**–**i**) Asci; (**j**–**m**) Ascospores. Scale bars, (**c**) 0.5 mm; (**d**) 100 μm; (**e**) 20 μm; (**f**–**i**) 10 μm; (**j**–**m**) 5 μm. Microscopic techniques: DIC.

***Phyllachora jiaensis*** H. X. Wu & J. C. Li. sp. nov. (Figure 4 and Figure 7).

MycoBank: MB 843398.

*Etymology:* Epithet derived from the type locality, Jia County (Shaanxi Province, Yulin City) in China.

Holotype: IFRD9448.

Parasitic on leaves and stems of *Chloris virgata* (Poaceae). Sexual morph: Stroma 825–2321 × 347–640 μm diam. ($\bar{x}$ = 1372 × 501 μm, *n* = 10) (Figure 7a–c), domed above the leaf surface, amphigenous, fusiform, cymbaeform or of irregular shape, black spots, scattered, sometimes gregarious, without an edge, shiny black, carbonaceous. Section of stroma 143–170 μm high, oval, multilocular, peridium 18–27 μm wide, composed of brown to dark-brown cells of textura angularis (Figure 7d). Paraphyses 3–4 μm wide ($\bar{x}$ = 3.1 μm, *n* = 20), numerous, persistent, filiform, unbranched, aseptate, slightly longer than asci (Figure 7e). Asci 77–114 × 8–12 μm ($\bar{x}$ = 93.4 × 9.4 μm, *n* = 20), thin-walled, 8-spored, persistent, clavate, apex obtuse, with pedicels (Figure 7f–i). Ascospores 9–17 × 8–9 μm ($\bar{x}$ =13.4 × 6.5 μm, *n* = 20), 1-seriate, ovoid to oblong, acute at both ends, hyaline, aseptate, verrucose, with 1–2 or more guttules (Figure 7j–m) and a glutinous mucilaginous sheath (Figure 4m–p). Asexual morph: Not observed.

Material examined: China, Shaanxi Province, Yulin City, Jia County, 38.02329299 N, 110.4956 E, on stems and leaves of *Chloris virgata* (Poaceae), 6 October 2019, Yuying Li, IFRD9448, holotype. GenBank accession numbers: LSU: ON075440, SSU: ON072100, ITS: ON075527.

Notes: In the course of investigating the grass resources of northern China, *Phyllachora jiaensis* was collected from Shaanxi Province. According to phylogenetic analysis, *P. virgatae* and *P. jiaensis* are closely related; however, they were collected from different locales. Morphologically, the stromata color of *P. virgatae* is bright black, and in *P. jiaensis* it is black. In addition, the asci of *P. virgatae* were longer than in *P. jiaensis* (102.6 × 9.1 μm vs. 93.4 × 9.4 μm). The ascospores of *P. jiaensis* were also longer than in *P. virgatae* (13.4 × 6.5 μm vs. 12.9 × 6.9 μm) (Table 2).

Phylogenetically, *P. virgatae* and *P. jiaensis* clustered together with high bootstrap support and probability value (100/1.0), with *P. jiaensis* forming a long branch. The LSU, SSU, and ITS loci differ by 8 bases, 109 bases, and 3 bases, respectively. Phylogenetically, *P. virgatae* grouped with *P. jiaensis* to form one clade, and *P. flaccidudis* with *P. sandiensis* to form another clade, with high bootstrap and probability values (100/1.0), but they occur on different hosts. *P. virgatae* and *P. jiaensis* both occur on *Chloris virgata*, and the host of *P. flaccidudis* and *P. sandiensis* is *Cenchrus flaccidus*. The ascospores of *P. flaccidudis* and *P. sandiensis* are acute at one end and blunt at the opposite end, while the ascospores of *P. virgatae* and *P. jiaensis* are blunt at both ends.

The four new species described herein have ascospores with gelatinous sheaths that differ in black ink (Figure 4). The gelatinous sheaths of *P. flaccidudis* and *P. sandiensis* are larger than in *P. virgatae* and *P. jiaensis*. Hence, based on both morphological and phylogenetic evidence, we introduce the novel species, *P. jiaensis*.

## 4. Discussion

In this study, we introduced four new taxa of *Phyllachora* (*P. flaccidudis*, *P. sandiensis*, *P. virgatae*, and *P. jiaensis*) that have morphological characteristics typical of *Phyllachora*: black leaf spots, peridium clypeate, multilocular, asci cylindrical, an unobvious apical ring, shortly pedicellate, numerous paraphyses and slightly longer than asci, and aseptate ascospores with guttules [4,33,50]. All novel taxa were introduced based on morphological characteristics and novel phylogenetic lineages in *Phyllachora* (Figure 1). We compared the morphological characteristics of the four new species and similar *Phyllachora* taxa (Table 2).

In recent years, several *Phyllachora* species have been introduced in many places of China, such as *Phyllachora heterocladae* (Sichuan, China), *P. panicicola* (Yunnan, China), *P. eriochloae* var. *colombiensis* (Yunnan, China), *P. graminis* var. *cynodonticola* Speg. (Yunnan, China), and *P. eriochloae* Speg. var. *eriochloae* (Yunnan, China) [20,33,43,44,45,46,47,48]. However, relevant molecular data only exists for a few of these species (e.g., *P. heterocladae* and *P. panicicola*). The majority of these species were reported from Yunnan Province (e.g., *P. panicicola*). Host specificity plays an important role when introducing novel *Phyllachora* species [3,4]. Yang et al. [20] proposed that phyllachora-like species that are parasitic on Poaceae should be treated as *Phyllachora*, and our study also provides strong evidence that supports this hypothesis.

Yang et al. [20] introduced *Phyllachora heterocladae* from Sichuan Province, and the phylogenetic tree was artificially divided into five lineages based on the host plants. Most species of *Phyllachora* that cluster within lineage I are graminicolous (Poaceae), but *P. qualeae* grows on *Qualea multiflora* (Vochysiaceae). They formed a distinct subclade with *P. arundinellae* (MHYAU:108), *P. cynodontis* (MHYAU:20043), and *P. imperatae* (MHYAU:014). Species within lineage II and lineage IV are bambusicolous fungi. Lineage III is solely composed of *P. thysanolaenae* (MFLU 16-2071), which is an unstable species in the phylogeny. Lineage V contains only *P. pomigena*, associated with an unknown host plant. Li et al. [32] introduced two new species, *P. dendrocalami-membranacei* and *P. dendrocalami-hamiltonii*, and phylogenetic analysis was generated four main clades. Lineage I consisted of all *Phyllachora* species obtained from the subfamily Agrostidoideae of the Poaceae, except for *Polystigma pusillum* (MM-19), which was found growing on Fabaceae. *Neophyllachora* species occurred in the family Myrtaceae within Lineage II. Lineage III and Lineage IV are *Phyllachora* species collected from the subfamily Bambusoideae of the Poaceae. However, it is important to note that Yang et al. [20] did not include all *Phyllachora* species in their analysis.

In this study, the generated phylogenetic tree comprises 74 species belonging to six genera *(viz*., *Ascovaginospora*, *Camarotella*, *Coccodiella*, *Neophyllachora*, *Phyllachora*, and *Polystigma*). We found that the *Phyllachora* genus is paraphyletic. Because the host of *P. pomigena* remains unknown, the species *Phyllachora pomigena* formed a single clade [20,51]. In the phylogenetic analysis, the new species described herein are included within the *Phyllachora* genus and separated from other taxa with a single subclade. Their hosts are *Cenchrus flaccidus* and *Chloris virgata*, both belonging to Poaceae (graminicolous).

The study revealed that the ancestor of *Phyllachora* species originated from Latin America. *Phyllachora* species ancestors initially spread from Latin America to North America, East Asia, South America, and eventually to Central Europe. The characteristic of *Phyllachora* species in Latin America are consistent with the ancestral characteristics of *Phyllachora* genus found in Mardones et al. [8]. For example, existing species *P. maydis* and *P. graminis* still retain ancestral characteristics, such as growing on monocotyledonous hosts, immersed perithecia, black stromata, and the presence of clypeus [8]. Reconstruction analysis of ancestral location indicates that a vicariance event (i.e., the splitting of the range of a taxon or biota into two or more geographical subdivisions by the formation of natural barriers, for example, mountain building, glaciation, plate tectonics or climate change) affected speciation allowing some species to retain ancestral morphological characteristics [52]. The appearance of *Polystigma* could have resulted from the extinction event (Figure 2 node 143). The extinction event may have resulted in the host of *Polystigma* species shifting from monocotyledons to dicotyledons (Fabaceae).

During the Cretaceous geological upheaval, orogeny, continental drift as well as the emergence of the Atlantic and the Indian Ocean led to dramatic terrestrial climate changes across the earth’s surface [53]. These led to the mass extinction of the dominant Mesozoic gymnosperm and ferns in the tropics, subtropical plains, and low mountains areas, which were replaced by angiosperms (the origin of Poaceae) that flourished in the Paleogene [54]. The emergence of angiosperms may have triggered the evolution and migration of the ancestors of the *Phyllachora* fungi.

There are few studies examining the co-evolution and ancestral state reconstruction of *Phyllachora* species; this is because of the scarcity of existing species with high-quality molecular data, which adds uncertainty to the process of ancestral state reconstruction. Extensive sampling and high-quality molecular data will reveal more accurate changes in the ancestral status of species in this group. Ancestor state reconstruction currently requires inferring phenotypes of ancestral species using observations from present-day species [55,56]. As new classical and molecular methods for identifying fungi continue to develop [57], ancestor state reconstruction analysis of fungal taxonomy is at the forefront of a new trend [8,58,59,60]. Future studies on species diversity and evolution of *Phyllachora* species require more extensive sampling and high-quality molecular data.

## Figures and Tables

**Table 1:** List of source and GenBank accession numbers used in this study. Sequences generated in this study are written in blue. Type collections are marked with ‘**^T^**’.

Species	Location	Source	Host Family	GenBank Accession Numbers			Reference
				LSU	SSU	ITS	
*Ascovaginospora stellipala* **^T^**	North America (northern Wisconsin)	P5-13A	Cyperaceae	U85088	U85087	-	[33]
*Camarotella costaricensis*	Latin America (Panama)	MM-21	Arecaceae	KX430490	KX451851	KX451900	[8]
*Camarotella costaricensis*	Latin America (Panama)	MM-149	Arecaceae	KX430484	KX451863	KX451913	[8]
*Camarotella* sp.	Latin America (Panama)	MM-27	Arecaceae	KX430492	KX451852	KX451901	[8]
*Coccodiella calatheae* ^**T**^	Latin America (Panama)	MP5133	Marantaceae	MF460370	MF460376	MF460366	[8]
*Coccodiella melastomatum*	North America (Venezuela)	CMU78543	Melastomataceae	-	U78543	-	[8]
*Coccodiella miconiae*	Latin America (Panama)	ppMP1342	Melastomataceae	KX430506	KX451871	MF460365	[8]
*Coccodiella miconiicola*	Latin America (Panama)	TH-571	Melastomataceae	KX430512	KX451880	-	[8]
*Coccodiella miconiicola*	Latin America (Panama)	CBMAP-H290A	Melastomataceae	MF460373	MF460379	MF460368	[32]
*Coccodiella miconiicola*	Latin America (Ecuador)	SO-15	Melastomataceae	MF460374	MF460380	MF460369	[32]
*Coccodiella toledoi*	Latin America (Ecuador)	MM-165	Melastomataceae	KX430488	KX451865	KX451917	[8]
*Neophyllachora cerradensis*	Latin America (Brazil)	UB21823	Myrtaceae	-	-	KC683470	[18]
*Neophyllachora cerradensis* ^**T**^	Latin America (Brazil)	UB21908	Myrtaceae	-	-	KC683471	[18]
*Neophyllachora myrciae*	Latin America (Brazil)	UB21292	Myrtaceae	-	-	KC683463	[18]
*Neophyllachora myrciae*	Latin America (Brazil)	UB22192	Myrtaceae	-	-	KC683476	[18]
*Neophyllachora myrciariae* ^**T**^	Latin America (Brazil)	UB21781	Myrtaceae	-	-	KC683469	[18]
*Neophyllachora subcircinans*	Latin America (Brazil)	UB09748	Myrtaceae	-	-	KC683441	[18]
*Neophyllachora subcircinans*	Latin America (Brazil), South America (Paraguay)	UB21347	Myrtaceae	-	-	KC683466	[18]
*Neophyllachora subcircinans*	Latin America (Brazil), South America (Paraguay)	UB21747	Myrtaceae	-	KC902622	KC683467	[18]
*Neophyllachora truncatispora*	Latin America (Brazil)	UB14083	Myrtaceae	-	KC902614	KC683448	[18]
*Phyllachora arthraxonis*	East Asia (China)	MHYAU:072	Poaceae	MG269803	-	MG269749	[20]
*Phyllachora arundinellae*	East Asia (China)	MHYAU:108	Poaceae	MG269815	-	MG269761	[20]
*Phyllachora capillipediicola*	East Asia (China)	MHYAU 20089	Poaceae	MG356698	-	KY498084	[32]
*Phyllachora capillipediicola*	East Asia (China)	MHYAU:20090	Poaceae	MG356699	-	KY498115	[20]
*Phyllachora chloridis* ^**T**^	Southeast Asia (Thailand)	MFLU 15-0173	Poaceae	MF197499	MF197505	KY594026	[33]
*Phyllachora chloridis*	Southeast Asia (Thailand)	MFLU 16-2980	Poaceae	MF197500	MF197506	KY594027	[33]
*Phyllachora chloridis-virgatae*	East Asia (China)	MHYAU 20136	Poaceae	MG356685	-	KY498122	[32]
*Phyllachora chloridis-virgatae*	East Asia (China)	MHYAU:20058	Poaceae	MG356683	-	KY498102	[32]
*Phyllachora chloridis-virgatae*	East Asia (China)	MHYAU 20137	Poaceae	MG356686	-	KY498092	[32]
*Phyllachora chrysopogonicola* ^**T**^	Southeast Asia (Thailand)	MFLU 16-2096	Poaceae	MF372146	-	MF372145	[20]
*Phyllachora cynodonticola* ^**T**^	Southeast Asia (Thailand)	MFLU 16-2977	Poaceae	MF197501	MF197507	KY594024	[33]
*Phyllachora cynodonticola*	Southeast Asia (Thailand)	MFLU 16-2978	Poaceae	MF197502	MF197508	KY594025	[33]
*Phyllachora cynodontis*	East Asia (China)	MHYAU 20042	Poaceae	KY498080	-	KY471328	[32]
*Phyllachora cynodontis*	East Asia (China)	MHYAU:20043	Poaceae	KY498081	-	KY471329	[20]
*Phyllachora cynodontis*	East Asia (China)	MHYAU 20131	Poaceae	KY498079	-	KY471327	[32]
*Phyllachora dendrocalami-hamiltoniicola*	East Asia (China)	MHYAU 221	Poaceae	MK614118	-	-	[32]
*Phyllachora dendrocalami-membranacei*	East Asia (China)	MHYAU 220	Poaceae	MK614117	-	MK614102	[32]
*Phyllachora dendrocalami-membranacei*	East Asia (China)	MHYAU 222	Poaceae	MK614119	-	MK614103	[32]
*Phyllachora flaccidudis* ^**T**^	East Asia (China)	IFRD9445	Poaceae	ON072101	ON072097	ON075524	This study
*Phyllachora graminis*	North America (Canada)	DAOM 240981	Poaceae	-	-	HQ317550	[33]
*Phyllachora graminis*	Central Europe (Germany)	MM-166	Poaceae	-	KX451869	KX451920	[8]
*Phyllachora heterocladae* ^**T**^	East Asia (China)	MFLU 18-1221	Poaceae	MK296472	MK296468	MK305902	[20]
*Phyllachora imperatae*	East Asia (China)	MHYAU:014	Poaceae	MG269800	-	MG269746	[20]
*Phyllachora indosasae*	East Asia (China)	MHYAU 125	Poaceae	MG195662	-	MG195637	[32]
*Phyllachora isachnicola* ^**T**^	East Asia (China)	MHYAU:179	Poaceae	MH018563	-	MH018561	[20]
*Phyllachora isachnicola*	East Asia (China)	MHYAU:180	Poaceae	MH018564	-	MH018562	[20]
*Phyllachora jiaensis* ^**T**^	East Asia (China)	IFRD9448	Poaceae	ON075440	ON072100	ON075527	This study
*Phyllachora keralensis*	East Asia (China)	MHYAU:20082	Poaceae	MG269792	-	KY498106	[20]
*Phyllachora maydis*	North America (USA)	BPI 893231	Poaceae	-	-	KU184459	[33]
*Phyllachora maydis*	North America (Wisconsin)	BPI 910560	Poaceae	-	-	MG881846	[20]
*Phyllachora miscanthi*	East Asia (China)	MHYAU:167	Poaceae	MG195669	-	MG195644	[20]
*Phyllachora miscanthi*	East Asia (China)	MHYAU:157	Poaceae	MG195668	-	MG195643	[20]
*Phyllachora panicicola* ^**T**^	East Asia (China)	MFLU 16-2979	Poaceae	MF197503	MF197504	KY594028	[33]
*Phyllachora pogonatheri*	East Asia (China)	MHYAU:071	Poaceae	MG269802	-	MG269748	[20]
*Phyllachora pogonatheri*	East Asia (China)	MHYAU:070	Poaceae	MG269801	-	MG269747	[20]
*Phyllachora pomigena*	unknown	CBS 194.33	Unknown	MH866861	-	MH855410	[20]
*Phyllachora pomigena*	unknown	CBS 193.33	Unknown	MH866860	-	MH855409	[20]
*Phyllachora qualeae*	unknown	UB 21159	Vochysiaceae	-	-	KU682781	[33]
*Phyllachora qualeae*	unknown	UB 21771	Vochysiaceae	-	-	KU682780	[33]
*Phyllachora sandiensis* ^**T**^	East Asia (China)	IFRD9446	Poaceae	ON075528	ON072098	ON075525	This study
*Phyllachora sinobambusae*	East Asia (China)	MHYAU 085	Poaceae	MG195655	-	MG195630	[32]
*Phyllachora sphaerocaryi* **^T^**	East Asia (China)	MHYAU 178	Poaceae	MK614114	-	MK614100	[32]
*Phyllachora sphaerocaryi*	East Asia (China)	MHYAU:178	Poaceae	-	-	MH018560	[20]
*Phyllachora thysanolaenae* **^T^**	Southeast Asia (Thailand)	MFLU 16-2071	Poaceae	-	MF372147	-	[20]
*Phyllachora virgataes* ^**T**^	East Asia (China)	IFRD9447	Poaceae	ON075439	ON072099	ON075526	This study
*Phyllachora yushaniae-falcatiauritae*	East Asia (China)	MHYAU 123	Poaceae	MG195656	-	MG195631	[32]
*Phyllachora yushaniae-polytrichae*	East Asia (China)	MHYAU 122	Poaceae	MG195657	MH992455	MG195632	[32]
*Phyllachora yushaniae-polytrichae*	East Asia (China)	MHYAU 158	Poaceae	MG195658	-	MG195633	[32]
*Polystigma pusillum*	Latin America (Costa Rica)	MM-113	Fabaceae	KX430474	KX451858	KX451907	[8]
*Polystigma pusillum*	Latin America (Costa Rica)	MM-147	Fabaceae	KX430483	KX451862	-	[8]
*Polystigma pusillum*	Latin America (Panama)	MM-19	Fabaceae	KX430489	KX451850	KX451899	[8]
*Polystigma* sp.	Latin America (Ecuador)	MM-163	Poaceae	KX430487	KX451864	KX451916	[8]
*Telimena bicincta*	Latin America (Costa Rica)	MM-108	Picramniaceae	KX430473	KX451857	KX451906	[8]
*Telimena bicincta*	Latin America (Costa Rica)	MM-133	Picramniaceae	KX430478	KX451861	KX451910	[8]

**Table 2:** Morphological comparison of four new species (in bold) and related species in *Phyllachora* reported from Poaceae.

Fungal Taxa	Hosts	Color of the Stromata	Asci (μm)	Ascospores (μm)			References
				Size	No. of Septa	Shape	
*P. africana* (*P. oblongospora*)	*Eremopogon delavayi*, *Leea elata*, *Chloris* sp.	Black	100–140 × 9–12.5	10–17 × 5–9	Aseptate	ovoid	[44]
*P. sphaerosperma* (*P. cenchricola*)	*Cenchrus echinatus*	Black	65–100 × 10–13	8–11 × 7–9	Aseptate	nearly spherical	[14]
*P. centothecae*	*Centotheca lappacea*	Bright	46.3 × 9.0, pedicel 11.6 × 2.6	7.7–9.0 × 4.6–5.1	Aseptate	oval	[45]
*P. coorgiana*	*Coix lachryma-jobi*	Bright	59–100 ×18–26	10.3–20.6 × 7.2–12.9	Aseptate	ellipsoid or ovoid, rarely subglobose	[45]
*P. chloridis*	*Chloris* sp.	Bright	50–72 × 6–8	8–12 × 3.5–4.8	Aseptate	fusiform to oval	[33]
*P. cynodontis*	*Chloris* sp.	Unknown	45–50 × 12–15 with a stipe 20–25 long, sometimes short	8–15 × 5–6	0–1	ovoid	[14,46]
*P. digitariicola*	*Digitaria sanguinalis*	Bright	62–103 × 12–15	10–18 × 6–8	Aseptate	ellipsoid, rounded at both ends, rarely subglobose or oval	[45]
*P. eriochloae* var. *colombiensis*	Gramineae	Unknown	37–71 × 102–13	4–13.9 × 5.4–7.2	Aseptate	ovate, rarely subglobose, rarely irregularly	[47]
*P. eriochloae* var. *eriochloae*	*Eragrostis* sp.	Bright	43.7–69.4 × 10.3–12.9	7.7–12.9 × 5.1–5.1	Aseptate	ovoid or tear-like	[47]
***P. flaccidudis***	***Cenchrus flaccidus***	**Black**	**82–110 × 7–10**	**11–13 × 4–7**	**Aseptate**	**drop shape, oval to ellipse, rounded at the ends**	**This study**
*P. graminis*	*Chloris* sp., *Elymus* sp., *Agvopyron* sp., *Arrenathevum* sp., *Asperella* sp., *Agrostis* sp., *Brachyelytvum* sp., *Bvomus* sp., *Cinna* sp.	Dark brown to black	60–70× 8–10	7–14× 4–7	Aseptate	oval to ovoid or ovoid with obtuse end flattened or blunted	[14]
*P. graminis* var. *cynodonticola*	*Cynodon dactylon*	Bright	82–87 × 7.7–8.1, with short peduncle 26 × 2.1	7.5–14 × 5.1–6.5	Aseptate	usually oblique, rarely irregularly biseriate, ellipsoid or subglobose	[47]
***P. jiaensis***	***Chloris virgata***	**Black**	**77–114 × 8–12**	**9–17 × 8–9**	**Aseptate**	**oval to ellipse, rounded at the ends**	**This study**
*P. koondrookensis*	*Chloris truncatae*	Black	75-87 × 12-16	14–16.5 × 5–5.5	Aseptate	uniseriatae vel inordinatae, anguste ellipsoideae usque and oblongae	[14]
*P. minutissima*	*Pennisetum flaccidum*, *Chloris* sp., *Panicum* sp., *Pennisetum* sp., *Pseudoechinochlaena* sp., *Setaria* sp.	Black	51.2–54.0 × 11.6–14.9	15.7–19.1 × 6.3–8.2	Aseptate	ovoid or ovate acuminately	[14,48]
*P. platyelliptica*	*Themeda giguntia*	Bright	64.1–92.1 × 10.1–12.1	13.6–16.5 × 3.8–6.5	Aseptate	narrow-ellipsoid	[48]
*P. panicicola*	*Panicum* sp.	Bright	110–130 × 10–14	14–16 × 6–8	Aseptate	ellipsoidal, rounded at the ends	[33]
***P. sandiensis***	***Cenchrus flaccidus***	**Black**	**92–126 × 7–10**	**10–14 × 6–7**	**Aseptate**	**drop shape, oval to** **ellipse**	**This study**
***P. virgatae***	***Chloris virgata***	**Bright**	**84–120 × 7–11**	**10–15 × 6–9**	**Aseptate**	**oval to ellipse, rounded at the ends**	**This study**

## Data Availability

All sequence data are available in NCBI GenBank following the accession numbers in the manuscript. All species data are available in MycoBank.
